# Patient-centeredness and determinant factors of palliative care service for adult cancer patients in public hospitals of addis Ababa, Ethiopia, 2024: cross-sectional mixed method study

**DOI:** 10.1186/s12904-025-01694-6

**Published:** 2025-03-07

**Authors:** Muday Beneberu, Getachew Teshale, Kaleb Assegid Demissie, Endalkachew Dellie, Melak Jejaw, Asmamaw Atnafu

**Affiliations:** 1 Department of Health Systems and Policy, Institute of Public Health, College of Medicine and Health Sciences, University of Gondar, Gondar, Ethiopia; 2 University of Gondar, Gondar, Ethiopia

**Keywords:** Palliative care, Cancer, Patient-centeredness, Addis Ababa, Ethiopia

## Abstract

**Introduction:**

Providing patient centered palliative care is essential to enhance the wellbeing of patients with life-limiting illnesses and their families. As the demand for palliative care services increases and the cancer burden grows in Ethiopia, it is crucial to know how much these services are patient centered and what factors may determine it. Therefore, this study assess the level and determinant factors of patient-centeredness for adult cancer patients’ palliative care services in public hospitals found in Addis Ababa.

**Method and materials:**

A cross-sectional mixed method was employed from May 16 to August 19, 2024. A total of 407 adult cancer patients, 7 key informant interviews and five in-depth interviews were included. The quantitative data was collected using Kobo collect tool version 2.023.21 and exported to STATA version 14 for analysis. Binary and multi-variable logistic regression with 95% Confidence Interval (CI) and Adjusted Odds Ratio (AOR) were fitted to identify factors associated with the patient-centeredness of care. Qualitative data was recorded, transcribed and thematically analyzed by using Open Code software version 4.0.1.

**Results:**

Patient-centeredness care for adult cancer patients was 77.9%. age group 30–39 (AOR: 3.52, 95% CI: 1.21, 10.21), being divorced (AOR: 0.14 95%, CI: 0.06, 0.37), monthly income > 12,000 Birr (AOR: 0.36, 95 CI: 0.156, 0.836), health literacy (AOR: 0.08, 95% CI: 0.02, 0.25), intimacy with the provider (AOR: 0.14 95% CI: 0.02, 0.75), service easiness (AOR: 0.34, 95% CI: 0.17, 0.67), and appointment waiting time (AOR: 0.4 95% CI: 0.19, 0.83) were found to be significant factors for patient-centeredness of palliative care service.

**Conclusion and recommendations:**

The magnitude of patient-centered care for adult cancer patients in palliative care was 77.9%. Most patients felt respected and involved in decision-making, and their physical and emotional comfort was maintained. However, only one-third of them reported ease of access to services. Age, marital status, income, participation in decision-making, intimacy with providers, ease of access, and appointment length was the significant factors. Recommendations include improving service coordination, reducing waiting time, fostering emotional connections between patients and providers, and offering tailored support to single or widowed patients to enhance patient-centered care.

## Introduction

Palliative care is the prevention and relief of the suffering of patients and their families. It is an approach that addresses the difficulties of having a life-threatening illness like cancer and easing pain and suffering [1]. The service includes diagnosing, treating, and meeting with the physical, spiritual, cultural, and religious needs of patients. It’s not an alternative to disease prevention and treatment, yet it is a service that should be integrated with them [2, 3].

Cancer is an abnormal cell division, uncontrollably and destruction of normal body tissues [4]. It is a huge public health burden and is the leading cause for morbidity and mortality worldwide. About 19.3 million new cases and 10.3 million deaths related to cancer worldwide by 2019/20 [5]. The burden on low and middle income countries (LMICs) is becoming disproportionate [6]. According to The Lancet Oncology report, in 2022, Ethiopia encountered 80,034 new cases and 54,698 deaths related to cancer [7].

The global increase in cancer incidence is making palliative care necessary for patients [8]. A study conducted in 2023 reported that people with cancer compromise about 34% of the population that needs palliative care worldwide [9]. According to a study conducted in all tertiary hospitals in Thailand, 7.8% of cancer patients required palliative care [10]. Almost all cancer patients in LMICs need palliative care. This makes their chance of experiencing severe pain and distress high [11].

Ethiopia is also one of the low- and middle-income countries with high burden of non-communicable diseases including cancer. Although most cancer patients need the palliative care services, only few of them received it due to limited access to opioid analgesia, and a concurrent lack of knowledge regarding the palliative care need [12]. A study done in a public hospital in Addis Ababa reported that 10.6% cancer patients required palliative care [13]. Another study conducted in a tertiary hospital in Addis Ababa, Ethiopia, also reported that among admitted cancer patients, 65% of them required palliative care service but didn’t receive any form of pain management [14].

Palliative care is an approach to care that is given to address the patient as whole, not just the disease [4]. Access to quality palliative care is considered as a basic human right. It needs to be delivered via person-centered, integrated health services that give careful consideration to each person’s unique requirements and preference.

Providing quality palliative care service is essential for improving the overall wellbeing of patients with life-limiting illness and their families [15]. There are multi-faceted challenges to the provision of quality palliative care. Some of these are: lack of awareness of healthcare providers, communities, and policy makers about the benefits of palliative care service. This will hinder the integration of the service into healthcare services [15]; inadequate resources, such as funding, drugs, trained professionals, etc [16].; financial and human resource constraints, weak collaboration among stakeholders; poor compliance of healthcare providers to the national guideline; poor holistic approach. Cancer patients get poor psychological, emotional, and physical care [16].

Patient-centered palliative care intends to make the whole person visible, be supported to live a life with dignity, and ultimately improve the QoL of patients and their families [17]. However, patients or their families were not engaged to make decisions about their care to enhance their quality of life, physical function, patient satisfaction, and quality of care (QOC) [18].

A national survey done in the US reported that only 21% of the institutions provided palliative a patient-centered care [19]. Another study done in Canada also revealed that only 71.6% of cancer patients perceived that they are adequately involved in decisions about their care [20]. A descriptive, cross-sectional study done in Kenya also found that 57.3% of cancer patients thought that the service they received was patient-centered [21]. A facility-based cross-sectional study done in Addis Ababa also showed that 37.2% cancer patients were not satisfied with the palliative care service [22].

Studies showed that palliative care services provided for cancer patients were not patient centered due to different challenges like lack of trained professionals, lack of opioids in health facilities, unwillingness of patients and their families to be referred to dedicated palliative care facilities [23]. Poor patient-centered care and lack of integrated and comprehensive palliative care can result in poor quality of life (QoL) [24]. This study aims to determine the level of patient centeredness of palliative care service for cancer patients and updating its determinant factors.

## Conceptual framework

**Fig. 1:**
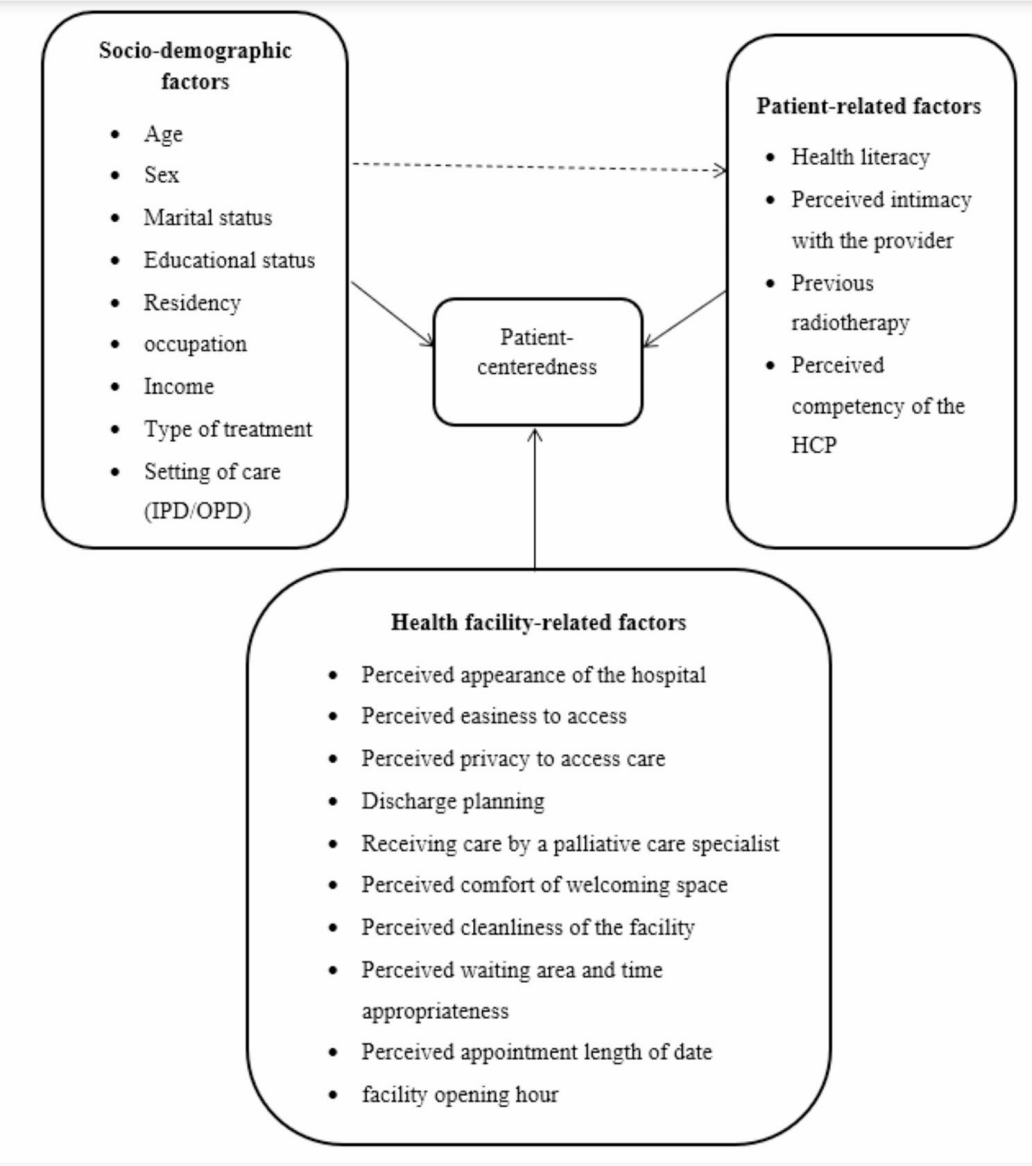
Conceptual framework for the evaluation of the quality of adult cancer patients’ palliative care services in public hospitals in Addis Ababa, Ethiopia, 2024 [25–29]

The conceptual framework identifies patient-centeredness as influenced by three key factors: socio-demographic, patient-related, and health facility-related. Socio-demographic factors (age, sex, marital status, education, income, etc.) shape patient experiences, while patient-related factors (health literacy, provider intimacy, radiotherapy experience, and provider competency) impact engagement and satisfaction. Health facility-related factors, such as hospital appearance, access, privacy, discharge planning, cleanliness, and waiting times, affect how patients perceive and interact with the healthcare facility. Together, these elements influence the overall patient-centeredness of care (Fig. 1).

## Method and design

### Study area and period

This study was conducted in public hospitals of Addis Ababa city administration from May 16 to August 19, 2024. Addis Ababa is the capital city of Ethiopia and have 11 sub-cities and 120 woredas (the lowest administrative units in the city). In the city administration, there are 14 public hospitals of which, Saint Paulos Millennium Medical Collage (SPMMC), Tikur Anbessa.

specialized hospital, Yekatit 12 and Zewuditu referral hospitals provided oncology services. Despite these four hospitals all provided palliative care services for all cancer and other life-threatening cases, Tikur Anbessa specialized hospital is the only center for radiotherapy treatment.

### Study design and method

This study employed a facility-based cross-sectional design utilizing explanatory concurrent mixed approach. Both quantitative and qualitative data were collected simultaneously (concurrently). The quantitative findings were explained and clarified with the qualitative results. The research was conducted in public hospitals in Addis Ababa, targeting adult cancer patients. The quantitative component aimed to assess the patient centeredness of through structured surveys, while the qualitative component explored challenges and limitations to provide patient centered care service for adult cancer patients using key informant interview.

### Populations and sampling

The source population for this study were all adult cancer patients receiving palliative care in public hospitals of Addis Ababa city administration. Case managers and palliative care unit coordinators were also included. All adult cancer patients who have been receiving palliative care for at least a month during the data collection period were included while patients who were critically ill excluded in our sampling.

### Sample size determination and sampling procedure

Since to the extent of our searching there was no studies done in the study area, we considered 50% population proportion, 10% non-response, 95% CI and 5% margin of error to calculate the sample size. Accordingly, 423 cancer patients who received palliative care service were included in the survey. The qualitative sample size was determined by the information saturation principle. Seven key informant interviews and five in-depth interviews were included.

The calculated sample size was proportionally allocated Tikur Ambesa specialized hospital, Siant Paulos Millennium Medical Collage (SPMMC), Yekatit 12, and Zewuditu referral hospitals based on the number of adult cancer patients under palliative care service unit month prior to the data collection period. A consecutive sampling technique was employed to recruit participants in each hospital until the required sample size is reached. The index (first) participant on the first day of the data collection period was selected randomly. Key informants (palliative care coordinators and case managers) were selected through purposive sampling. This allowed obtaining rich information from those who had a better understanding, experience, and knowledge regarding cancer palliative care services. The in-depth interview participants also selected purposively based on their length of admission. Accordingly, five adult cancer patients who were on palliative care for three months were included.

### Variables, indicators and their measurement

#### Dependent variable

The dependent variable is the patient-centeredness of palliative care services for adult cancer patients. It was measured using a 21-item, five-point Likert scale structured questionnaire and 8 indicators. The outcome variable was dichotomized as either “good patient-centered care” or “poor patient-centered care,” based on the demarcation threshold formula with a cut-off point of 83 [30]. The cut-off point was calculated as:

$${\rm{Cut - off}}\,{\rm{point = }}\,{\matrix{{\rm{(total}}\,{\rm{highest}}\,{\rm{score}}\,{\rm{ - }}\,{\rm{total}}\,{\rm{lowest}}\,{\rm{score}}\, \hfill \cr{\rm{ + }}\,{\rm{total}}\,{\rm{lowest}}\,{\rm{score)}} \hfill \cr} \over {\rm{2}}}$$

Accordingly, responses with a score of 83 and above were categorized as “good patient-centered care,” while those below were categorized as “poor patient-centered care.”

#### Independent variables

The independent variables include socio-demographic factors and clinical characteristics such as age, sex, marital status, educational status, health literacy, residency, occupation, income, type of treatment, and care setting (inpatient/outpatient). Patient-related factors include involvement in decision-making and treatment planning, perceived intimacy with the provider, prior radiotherapy, and perceived competency of the healthcare provider. Organizational-related factors include perceived hospital appearance, ease of access, privacy in accessing care, communication of the care plan, receiving care from a palliative care specialist, perceived welcoming environment, cleanliness of the facility, waiting area and time, appointment scheduling, and opening hours.

##### Health literacy

it is defined as is the understanding of patients about their health condition and treatment options. It was measured by asking patients five yes or no item questions and a patient said to have health literacy if she/he answer three or more questions correctly [31].

### Indicators

The following indicators were developed from various literature sources and the Ethiopian National Palliative Care Guideline [32–36] to measure the patient-centeredness of palliative care services provided to adult cancer patients. These indicators include:

- Proportion of cancer patients who perceived that healthcare providers respected their preferences.
- Proportion of cancer patients who perceived that the palliative care provided ensured physical comfort.
- Proportion of cancer patients who perceived that the palliative care services were well-coordinated.
- Proportion of cancer patients who perceived good continuity and transitions in care.
- Proportion of cancer patients who perceived that they received emotional support.
- Proportion of cancer patients who perceived that the care they received was easily accessible.
- Proportion of cancer patients who perceived that they were well-informed and educated about their care.
- Proportion of cancer patients who perceived that their families and friends were encouraged to be involved in their treatment plan.

### Data collection tools and field work

An interviewer-administered structured questionnaire, which was adapted from a study done on patient-centeredness of health services in Addis Ababa [37], was used for patient interview to assess the patient-centeredness of palliative care service provided at the selected public hospitals in Addis Ababa. The questions were categorized into the four domains: socio-demographic and clinical characteristics of patients, 5- point likert scale labeled as “strongly agree”, “agree”, “neutral”, “disagree”, and “strongly disagree” with 21 items, health facility related factors with 11 “yes” or “no” questions, and patient-related factors with 4 “yes” or “no” questions.

The data collectors obtained verbal informed consent from the study participants before collecting the data. Six trained BSc nurse data collectors and two MPH-level supervisors were recruited trained for two days about the data collection process, data quality and ethical issues.

A semi-structured key informant interview and in-depth interview guide was adapted from the national guideline guide line [36]. The interviewer also used probing questions during the interviews. The principal investigator conducted the key informant and in-depth interviews.

### Data quality assurance

To ensure the consistency and completeness of the questionnaire prepared for assessing patient-centeredness, a pre-test among 5% of the sample size was carried out on cancer patients at the University of Gondar Comprehensive Specialized Hospital (UOGCSH). The reliability of the survey tool was checked by calculating Cronbach’s alpha and the result was 0.9021 with an average inter-item covariance of 0.287. The qualitative study experts had checked the validity (content validity) of qualitative tools. Before the data collection commenced, data collectors and supervisors were trained for two consecutive days. The supervisors and principal investigator checked the completeness and consistency of data daily. The electronic data was locked with a secure devise and protected from non-privileged manipulation.

The key informant and in-depth interviews were conducted in Amharic, and translated to English for analysis. The transcribed data was cross-checked against the field note and the translated data was taken back to some of the key informants and in-depth interview participants (member checking) to make sure if the transcribed data represented their views. Moreover, the transcripts were cross-checked with the audio files to ensure accuracy and consistency of the qualitative data before coding and analyzing.

### Data management and analysis

Data was collected using Kobo collect tool version 2.023.21, checked for completeness and consistency cleaned before analysis conducted. STATA version 14 was used for analysis of quantitative data. for further analysis. Descriptive statistics, including means, standard deviations, frequencies and percentages were calculated and presented in the form of narrations, tables and graphs. Binary logistic regression model was fitted to identify factors associated with patient-centeredness of care. Variables with a p-value of < 0.25 were candidates for multivariable analysis. In multivariable logistic regression analysis, the adjusted odds ratio (AOR) with a 95% confidence interval (CI) and *p* < 0.05 were used to declare statistical significance. A multi-collinearity test was employed for the model and the mean VIF was 2.09 with values ranging from 1.36 to 4.09. A multivariable logistic regression model was fitted with Hosmer and Lemeshow’s goodness of model fitness test (*p* = 0.2681) for patient centeredness of care.

Audiotaped data was transcribed verbatim to Amharic, and then it was translated into English. The translated data was coded and thematized by Open code version 4.01 software. Finally, the report was written meaningfully in ways participants say with a quotation.

## Results

A total of 407 study participants with a response rate of 96.2% responded for our survey. Besides 7 key informant and 15 in-depth interviews were conducted.

### Socio-demographic and clinical characteristics respondents

From the total respondents, 214 (52.6%) were females and 193 (47.4%) were males. The respondents’ mean age was 52.23 (SD ± 13.5) and most of them 266 (65.4%) lived in the urban residents. Over half of them were married 226 (55.5%), and 122 (29.9%) had completed secondary school. Over one-fourth of 115 (28.26%) of the respondents worked in the private company and 182 (44.72%) had 2001–7000 ETB monthly income (Table 1).

**Fig. 2:**
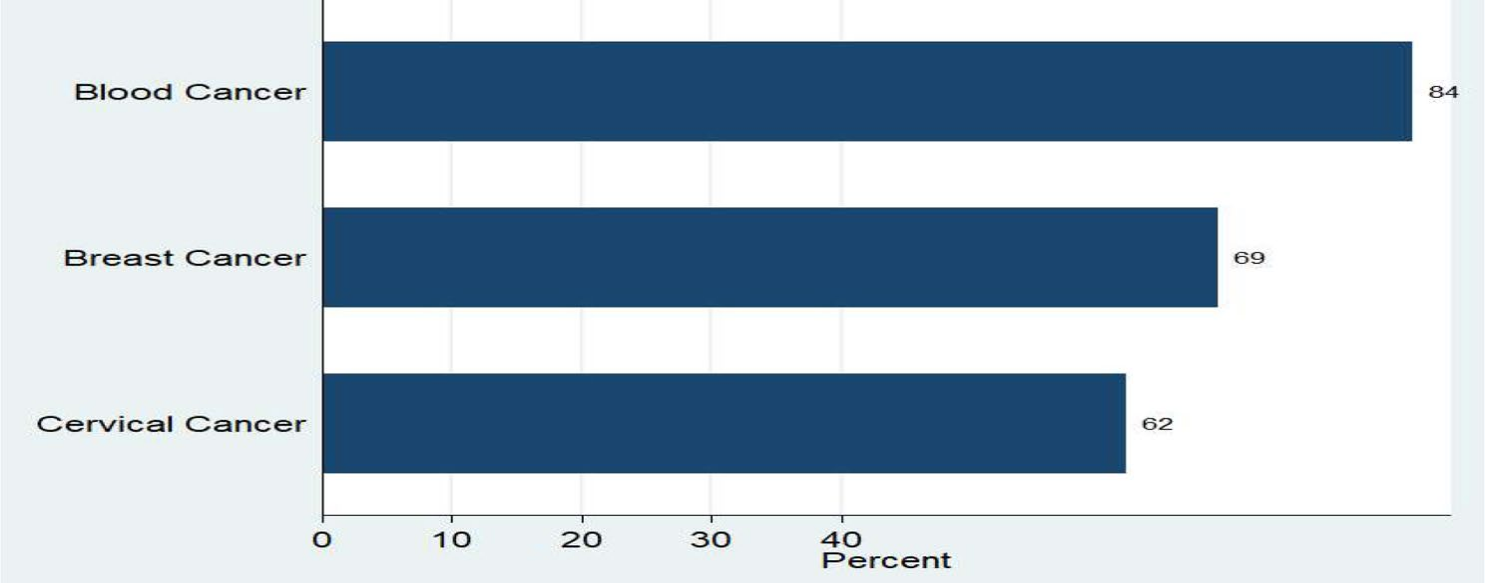
Graphical representation of the frequent cancer types among adult cancer patients on palliative care service in Addis Ababa public hospitals, Addis Ababa, Ethiopia, 2024 (*n* = 407)

### Respect for patients’ preferences

Majority (84.4%) of the study participants perceived that in the provision of palliative care they were treated with respect and dignity, they and their families were involved in management plan, actively participated in the development of a treatment plan with the providers, and the service provided was guided by the medical-ethical principles. The key informant interview also revealed this finding.

*Since cancer patients should not treat as others*,* the patients and their families participated in their treatment plan*,* schedule and overall management. Palliative care is not just about giving medicine*,* but starting from home; you have to take the history of what they have and solve it well*, *read them*,* their psycho and everything. [36 years old, female cancer unit case manager]*

Although this might be good, due to the high flow of patients in these hospitals, addressing the needs of these patients and providing a care that is tailored to their needs can be difficult. A case manager working in one of the public hospitals explained this problem as

I have worked here for 4 years and I have witnessed that high patient flow in the cancer treatment unit and respecting the needs of all these patients is very difficult. It would be better if we had more manpower than we have at the moment. This may help to improve the quality of the service and provide patient-centered care for these patients. [32 years old, female cancer unit case manager]

### Physical comfort

Majority of the participants (82.3%) agreed that attention was given to physical comfort such as, the management of pain, and shortness of breath. The key informant interview also revealed that:

All our patients are assessed for the severity of pain and pain score is recorded. Accordingly, sedatives and pain management provided. In principle, pain is considered as the fifth vital sign for all patients and we did it for all patients and when needed. [29 years old, female palliative care coordinator]

The main activity done in palliative care is symptom alleviation. For example, when a patient is being in respiratory distress, the fluid will be forced out of the lungs, and then the patient will be prescribed with tramadol or morphine. [38 years old, male cancer unit case manager]

### Coordination and integration of care

Most of the study participants (85.4%) perceived that the palliative care provided in the facilities was coordinated and integrated. One of the patient interviews also revealed this:

*“The doctors here are very good and they do their duty assignment. All the doctors and nurses provided necessary information about my health condition. They provide the service equally whatever the patient socio-economic status and for all wards. [37 years old, female, liver cancer patient]*

### Continuity and transition

Majority of (90.9%) the respondents agreed that the advice they received from different providers was well attuned to each other. The key informant interview with the service user also witnessed this.

Since the providers have expert knowledge, the advice they give us about our condition is not distorted and the way they provide the service is harmonious. [A 70 years old, female cervical cancer patient]

### Emotional support

The majority of the study participants (82.4%) perceived that the palliative care provider addressed their emotional and spiritual needs and felt that their hopes, fears, and expectations were addressed in the context of social and cultural norms.

*“A patient knows they are going to die and speaks to their creator. We have a counselor who provides daily counseling services. In addition*,* the doctors are trained to offer counseling*,* so they help support the patients’ emotional well-being every day. After one or two days of counseling*,* the patients feel better mentally and psychologically*,* even though their clinical condition may worsen.” [29 years old, female palliative coordinator]*

They [providers] help us to the best of their knowledge; they try to know about and understand our feelings, and take care of everything that is necessary. [58 years old, male, lung cancer patient]

### Access to care

Access to care was measured in terms of having an ease of access to laboratory results and medications, length of appointment time, and accessing diagnostic works on the same day. Over two-third 67.5% of the respondents reported that accessing palliative care service was not easy them and their families.

The key informant interview response revealed that accessing medications like morphine was a big challenge for inpatient service.

As you know the drug morphine is key to cancer patients. However, the drug was not available in the facility for long period of time. It wasn’t in stock for a very long time and now it is easy to say no morphine at all. [38 years old, male cancer unit case manager]

The in-depth interview with patients also showed that they faced difficulties to laboratory tests.

The problem is that it takes a lot of time to get laboratory results, and there is a lot of hassle. That is the biggest problem. [78 years old, female, ovarian cancer patient]

Besides, accessibility of cancer drugs in the public facilities and affordability of these drugs was a predicament for patients receiving chemotherapy.

*“The chemotherapy medicine has to be bought from outside*,* and it’s quite expensive. I spend 20*,*000 Birr for just 15 days. I’m okay because my children are supporting me*,* but there are people who have absolutely nothing. What should they do? Should they stop their treatment? If the medicine were available here or if the government had planned and made it accessible in this hospital*,* like with HIV treatment*,* it would make a difference. For example*,* no one misses out on HIV or diabetes medication because they’re essential. Now*,* cancer is becoming as dangerous as HIV and diabetes. Everyone should work to stop the spread of cancer. If the medicine were free and available in every hospital*,* and if more attention were given to cancer as it is with HIV*,* it would make treatment much easier.” [64 years old, female, gastric cancer patient]*

*“Medicines here are affordable only for those with health insurance. If you don’t have insurance*,* you can’t buy them here. We buy our medicine from outside because we don’t have health insurance. If the hospital sold the medicine to us at their prices*,* it would make a huge difference compared to buying it outside. Medicine is very expensive outside the hospital*,* but it’s much cheaper here. It’s great for those with insurance because they get a discounted price. For the rest of us*,* we buy the medicine elsewhere and bring it in*,* but nothing else is done for us regarding the cost of medicine. If the hospital sold it at this price or if the government provided it*,* the medicine could be used for two or three rounds of treatment.” [23 years old, female, breast cancer patient]*

Due to the large volume of patients and lack of sufficient treatment rooms, they may have to reschedule their appointment date. In our in-depth interview on patient explained this problem as:

*“It would be preferable to have more treatment rooms. Sometimes*,* when you come for an appointment*,* there’s a long queue*,* and you may have to wait until the third week*,* depending on how many people are there. The number of patients and the available rooms are not proportional. For example*,* there are patients who are being treated in beds*,* and waiting in line can lead to complications. It would be helpful if they could address this issue.” [23 years old, female, breast cancer patient]*

### Information and education

Most of the respondents (83.6%) agreed that they were informed about common neuropsychiatric issues, communicated about their treatment, and discussed on their diagnosis. They also perceived that they were served by a palliative care professional and educated and advised about nutrition and their health condition.

*“The doctors here in Black Lion Specialized Hospital are very good. They visited us and discuss the importance of diet*,* treatment*,* and exercise. The physicians here*,* along with those specialists*,* always explain what is happening and keep us informed.” [55 years old, female, ovarian cancer]*

### Involvement of family and friends

Involvement of family and friends assessed if attention was given to care and support provided by family members and possible questions from them. Majority of the respondents (87.4%) agreed that their families and friends were given an appropriate attention (Table 3).

They [the providers] give the necessary advice to family members; they respond in an appropriate manner whenever there is a question, and they also ask us what we feel and tell us how the service should be provided. [58 years old, male, lung cancer patient]

**Fig. 3:**
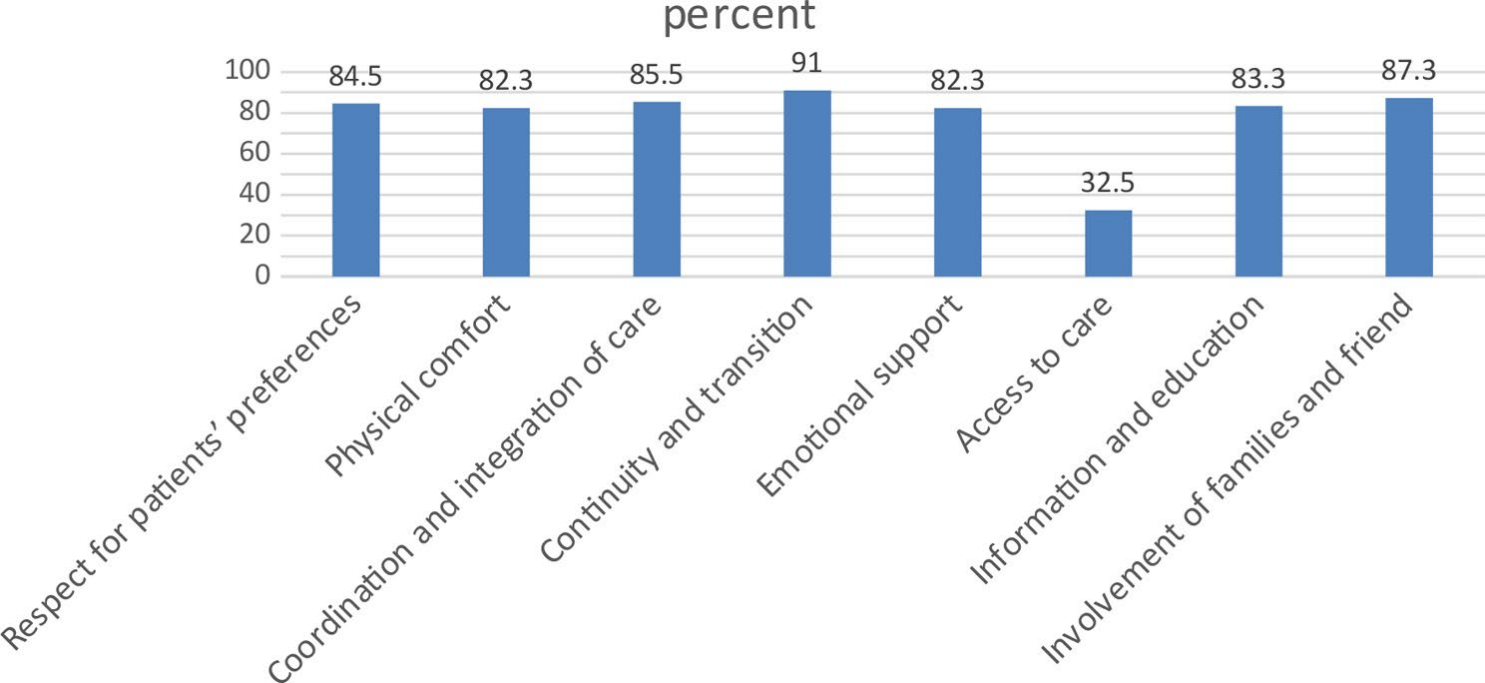
Indicators and patient perception on patient-centeredness palliative care services

### Factors of patient-centeredness of adult cancer patients in palliative care

Majority (89.43%) of respondents believed that they understood their conditions. Additionally, most participants (93.6%) reported that they and the physician treating them were familiar with each other and nearly all participants (97.8%) perceived that the physician treating them was knowledgeable about their condition. A smaller portion (21.62%) had previously undergone radiotherapy.

The majority of respondents, 375 (92.1%) felt that the hospitals had a welcoming environment and approach for patients and families. Of the total respondents, 255 (62.7%) found accessing services to be easy. Additionally, 308 (75.7%) perceived the external appearance of the hospitals as attractive. However, only 210 (51.6%) believed there was adequate privacy when receiving care. A large portion (90.42%) agreed that they received clear instructions on how to manage their care after leaving the hospital, and 95.6% confirmed they were treated by a palliative care specialist. Among the respondents, 304 (74.7%) thought the facility was clean, 298 (73.2%) found the waiting area comfortable, 211 (51.8%) did not experience long waits for services, and 249 (61.2%) felt their appointment time is not too long. Finally, 309 (75.9%) were satisfied with the hospital’s operating hours (Table 4).

**Table 1:** Socio-demographic characteristics of adult cancer palliative patients at addis Ababa public hospitals, addis Ababa, Ethiopia, 2024 (*n* = 407)

Variables	Category	Frequency	Percentage
Sex	Male	193	47.42%
	Female	214	52.58%
Age	<= 20 years	1	0.24%
	21–35 years	49	12.04%
	36–50 years	128	31.45%
	51–65 years	168	41.28%
	66–80 years	51	12.53%
	> 81 years	10	2.46%
Marital status	Single	72	17.7%
	Married	226	55.53%
	Divorced	60	14.74%
	Widowed	49	12.04%
Education	No education	80	19.7%
	Primary school	88	21.62%
	Secondary school	122	29.9%
	Higher education	117	28.8%
Residence	Urban	266	65.4%
	Rural	141	34.64%
Occupation	Government worker	102	25.1%
	Private worker	115	28.3%
	Merchant	70	17.2%
	Retiree	46	11.3%
	Other	74	18.2%
Monthly income	<= 2000 Birr	87	21.4%
	2001–7000 Birr	182	44.72%
	7001–12,000 Birr	73	17.9%
	> 17,000 Birr	65	15.9%

Most of the study participants 270 (66.34%) were on chemotherapy while 51 (12.53%) and 34 (8.3%) took radiotherapy and combined therapy (both chemotherapy and radiotherapy) respectively. Most of the study participants 281 (69.04%) were receiving palliative treatment in outpatient care and half of them 205 (50.4%) were on palliative care for three months (Table 2)

**Table 2:** Clinical characteristics of adult cancer patients on palliative care service in addis Ababa public hospitals, addis Ababa, Ethiopia, 2024 (*n* = 407)

Variables	Category	Frequency	Percentage
Time in care	1–3 months	205	50.4%
	4–6 months	129	31.7%
	7–12 months	70	17.2%
	> 12 months	3	0.74%
Setting of care	Outpatient	281	69.04%
	Inpatient	126	30.9%
Type of pain treatment	Chemotherapy	270	66.34%
	Radiotherapy	51	12.53%
	Chemotherapy and radiotherapy	34	8.4%
	Other	52	12.8%

Adult cancer patients received the palliative care service includes blood cancer 84 (20.64%), breast cancer 69 (16.9%), and cervical cancer 62 (15.23%) (Fig. 2)

**Table 3:** patient-centeredness of adult cancer patients in the palliative care services in public hospitals, addis Ababa, Ethiopia, 2024 (*n* = 407)

Variables	Indicator	Frequency	Percent (%)
Respect for patients’ preferences	Proportion of cancer patients who perceived that the HCPs respect their preferences	344	84.5
Physical comfort	Proportion of cancer patients who perceived that the palliative care provided ensures physical comfort	335	82.3
Coordination and integration of care	Proportion of cancer patients who perceived that the palliative care service is coordinated	348	85.5
Continuity and transition	Proportion of cancer patients who perceived the care has good continuity and transition	370	91
Emotional support	Proportion of cancer patients who perceived that they received emotional support	335	82.3
Access to care	Proportion of cancer patients who perceived that the care they receive is not easily accessible	275	67.5
Information and education	Proportion of cancer patients who perceived that they well informed and educated	340	83.3
Involvement of families and friend	Proportion of cancer patients who perceived that their families and friends were encouraged to involve in their treatment plan	356	87.3

As shown in the figure below the around 78% of the cancer patients received patient centered palliative care majority (91%) of the patients perceived the care has good continuity and transition while very few of them (32.5%) perceived that the care they receive is easily accessible (Fig. 3)

**Table 4:** Patient and health facility-related factors of adult cancer patients palliative care service in public hospitals of addis Ababa, Ethiopia, 2024 (*n* = 407)

Variable	Category	Frequency (*n* = 407)	Percentage (%)
Health literacy	Yes	364	89.43
	No	43	10.60
Intimacy with the provider	Yes	381	93.60
	No	26	6.40
Previously taken radiotherapy	Yes	88	21.62
	No	319	78.40
Physician knowledge	Yes	398	97.80
	No	9	2.20
Hospitality	Yes	375	92.10
	No	32	7.90
Privacy	Yes	210	51.60
	No	197	48.40
Cleanliness	Yes	304	74.70
	No	103	25.30
Ease of access	Yes	255	62.70
	No	152	37.40
Discharge plan	Yes	368	90.42
	No	39	9.60
Waiting area comfort	Yes	298	73.20
	No	109	26.80
Attractiveness	Yes	308	75.70
	No	99	24.32
Specialist care	Yes	389	95.60
	No	18	4.40
Waiting time	Yes	196	48.20
	No	211	51.80
Appointment waiting time	Yes	158	38.80
	No	249	61.20
Opening hour	Yes	309	75.90
	No	98	24.10

A binary logistic regression was performed on the independent variables, and those with a p-value < 0.25 were selected as candidates for multivariable logistic regression. These variables included: age, sex, marital status, education, income, care setting, participation, intimacy with the provider, ease of access, privacy, hospitality, communication of the treatment plan, cleanliness, waiting area comfort, attractiveness, waiting time, length of appointment time, and operating hours.

In the multivariable logistic regression, independent variables with a p-value < 0.05 were considered significantly associated with patient-centered care. These variables included age, marital status, monthly income, health literacy, intimacy with the provider, ease of access, and length of appointment time.

Accordingly, Patients with 30–39 age group were 3.52 times more likely (AOR: 3.52, 95% CI: 1.26, 10.21) to perceive the care as patient-centered compared to 50–59 age group. The odds of perceiving care as patient-centered decreased by 86% for divorced patients (AOR: 0.14, 95% CI: 0.06, 0.38) as compared to married patients. For those with a monthly income greater than 12,000 Birr, the odds of patient-centered care decreased by 64% (AOR: 0.36, 95% CI: 0.156, 0.84) as compared to those with an income between 2,001 and 7,000 Birr.

Patients who hadn’t health literacy were 99.2% less likely (AOR: 0.08, 95% CI: 0.02, 0.25; *p* < 0.001) to experience patient-centered care as compared to those who had health literacy. Patients who did not perceive intimacy with their provider were 86% less likely (AOR: 0.14, 95% CI: 0.02, 0.75) to get patient-centered care as compared to those who felt they had a close relationship with their provider.

The odds of getting patient-centered care was decreased by 66% for patients who faced difficulties to access the services (AOR: 0.34, 95% CI: 0.18, 0.67) as compared to their counters. Finally, patients who experienced long appointment waiting time were 60% less likely (AOR: 0.40, 95% CI: 0.19, 0.83) to perceive the service as patient-centered compared to those who did not wait long (Table 5).

**Table 5:** Bi-variable and multivariable analysis of factors associated with the patient-centeredness of adult cancer patients’ palliative care in public hospitals of addis Ababa, Ethiopia, 2024 (*n* = 407)

Variable	Category	Patient-centeredness		COR (95% CI)	*p*-value	AOR (95% CI)	*p*-value
		Yes	No				
Sex	Female	108	106	1.02 (0.779–1.332)	0.891	1	
	Male	82	111	0.73 (0.49–1.072)	0.107	0.69 (0.396–1.217)	0.202
Age of respondents	20–29	11	12	1.13 (0.464–2.765)	0.783	1.30 (0.330–5.169)	0.702
	30–39	38	13	3.61 (1.754–7.448)	0.000	3.52 (1.215–10.209)	0.020*
	40–49	39	59	0.82 (0.477-1.400)	0.463	0.83 (0.417–1.649)	0.593
	50–59	55	68	0.81 (0.513–2.353)	0.242	1	
	60–69	31	47	0.82 (0.458–1.450)	0.488	0.72 (0.322–1.599)	0.417
	≥ 70	16	18	1.09 (0.513–2.353)	0.808	0.84 (0.288–2.459)	0.752
Marital status	Married	109	117	0.93 (0.718–1.209)	0.595	1	
	Single	47	25	2.02 (1.163-3.500)	0.012	1.68 (0.709–3.995)	0.237
	Divorced	7	53	0.14 (0.062–0.325)	0.000	0.14 (0.055–0.375)	0.000*
	Widowed	27	22	1.32 (0.708–2.449)	0.384	1.35 (0.565–3.232)	0.498
Occupation	Private worker	44	71	0.62 (0.425–0.903)	0.013	1	
	Government worker	50	52	1.55 (0.904–2.664)	0.111	2.04 (0.924–4.519)	0.078
	Merchant	28	42	1.08 (0.585–1.977)	0.814	1.68 (0.745–3.809)	0.211
	Retiree	22	24	1.48 (0.742–2.949)	0.266	1.47 (0.481–4.467)	0.501
	Other	46	28	2.65 (1.452–4.839)	0.001	1.59 (0.687–3.701)	0.277
Monthly income of respondents (Birr)	≤ 2000	54	33	1.79 (1.061–3.011)	0.029	1.16 (0.559–2.416)	0.688
	2001–7000*	87	95	0.92 (0.685–1.225)	0.553	1	
	7001–12,000	29	44	0.72 (0.415–1.249)	0.243	0.74 (0.340–1.593)	0.437
	> 12,000	20	45	0.49 (0.266–0.886)	0.019	0.36 (0.156–0.836)	0.017*
Setting of care	Outpatient	115	166	2.12 (1.384–3.256)	0.002	1	
	Inpatient	75	51	0.69 (0.546–0.879)	0.001	1.42 (0.810–2.476)	0.222
Health literacy	Yes	185	179	1.03 (0.842–1.269)	0.753	1	
	No	5	38	0.13 (0.049–0.331)	0.000	0.08 (0.024–0.248)	**0.000**
Education	No education	41	39	0.95 (0.542–1.675)	0.866	0.97 (0.437–2.159)	0.943
	Primary school	36	52	0.63 (0.361–1.092)	0.099	0.58 (0.279–1.219)	0.152
	Secondary school	64	58	1.10 (0.773–1.574)	0.587	1	
	Higher education	49	68	0.65 (0.392–1.089)	0.102	0.49 (0.225–1.091)	0.081
Intimacy with the provider	Yes	188	193	0.97 (0.797–1.190)	0.001	1	
	No	2	24	0.09 (0.019–0.367)	0.798	0.14 (0.024–0.751)	0.022*
Hospitality	Yes	183	192	0.95 (0.778–1.167)	0.642	1	
	No	7	25	0.29 (0.124–0.696)	0.005	0.90 (0.271–3.009)	0.869
Ease of access to services	Yes	154	101	1.52 (1.186–1.959)	0.001	1	
	No	36	116	0.20 (0.129–0.319)	0.000	0.34 (0.177–0.671)	0.002*
Cleanliness	Yes	161	143	1.13 (0.899–1.410)	0.302	1	
	No	29	74	0.35 (0.214–0.565)	0.000	0.42 (0.158–1.103)	0.078
Waiting area comfort	Yes	156	142	1.09 (0.875–1.379)	0.418	1	
	No	34	75	0.41 (0.259–0.657)	0.000	1.58 (0.644–3.860)	0.318
Long appointment waiting time	Yes	42	116	0.25 (0.160–0.381)	0.000	0.40 (0.196–0.830)	0.014*
	No	148	101	1.47 (1.138–1.887)	0.003	1	
Long waiting time to get the service	Yes	70	126	0.42 (0.283–0.628)	0.000	1.18 (0.565–2.449)	0.663
	No	120	91	1.32 (1.004–1.732)	0.047	1	
Discharge plan	Yes	181	187	0.97 (0.789–1.187)	0.754	1	
	No	9	30	0.31 (0.143–0.671)	0.003	0.90 (0.285–2.862)	0.863
Opening hour	Yes	158	151	1.05 (0.837–1.308)	0.690	1	
	No	32	66	0.46 (0.287–0.747)	0.002	1.29 (0.610–2.726)	0.505
Attractiveness	Yes	159	149	1.07 (0.853–1.334)	0.569	1	
	No	31	68	0.43 (0.264–0.690)	0.001	0.93 (0.419–2.081)	0.866

*: refers significant variables in multi-variable logistic regression model with *p* < 0.05 and 95% CI

## Discussion

This study determined the level of patient centeredness of palliative care services for cancer patients and identified it’s determinant factors. The magnitude of patient-centeredness care for adult cancer patients in palliative care was found to be 77.9% (95% CI: 0.66–0.94). the finding was higher as compared to the previous study done in Bahr Dar city. The variation may be due to the previous study included both public and private facilities and stated that it was higher among public hospitals [49]. Although the majority (84.9%) of the respondents perceived that the palliative care service providers respected them and their families, the finding was lower than a recommendation stated as in that all palliative care service should be provided with respect to patients’ needs and individual preferences [40]. The reason for this deviation was stated in our qualitative part as:

*Due to the high flow of patients in the hospitals*,* addressing the needs of these patients and providing a care that is tailored to their needs can be difficult.*

It was also lower than an international survey conducted in 7 countries which was found 94% of end-stage cancer patients under palliative treatment responded that their needs and preferences were preserved and that the physicians treated them and their families with respect and dignity [38]. The possible explanation for this difference could be variation in sample size, the measurement tools used, and the difference in the study settings.

Even though our quantitative analysis showed that 82.3% of the respondents perceived that they had physical comfort during palliative care. This finding was found to be higher than the study done in Japan, in which 58% palliative cancer patients reported they had physical distress [39]. The possible reason for this difference might be the study period, as a patient-centered approach to address the needs and relief the pain for palliative patients has gained attention recently. The IOM recommended healthcare providers to swiftly address patients’ physical needs and symptoms, as well as provide them with adequate pain management [40]. However, the qualitative result revealed that:

*Despite morphine is key to cancer patients*,* the drug was not available in the facility for long period of time*.

Our study also showed that over 85% of the participants perceived that the palliative care was integrated and coordinated. This finding was higher than the study done in the US, which reported that 60.2% patients perceived that they had received a coordinated care [41]. This difference might be due to the difference in the study periods as currently there is a huge advancement in the provision of a coordinated and integrated palliative care service to cancer patients.

Most of our study participants perceived that they received emotional needs were effectively addressed in addition to their physical needs. Our qualitative finding also supported this result and one of the providers responded as:

We have a counselor who provides daily counseling services. In addition, the doctors are trained to offer counseling, so they help support the patients’ emotional well-being every day.

The finding was higher than the study done in Bangladesh, in which the perceived emotional support was 74.7% [42]. This difference might be due to the difference in sample size and study method employed.

In contrast with the IOM suggestion of facilities that provide palliative care services have to make sure that patients have timely access to the service they need [40], only one-third of our study respondents reported that accessing services like lab results and medicines was easy. The qualitative finding s revealed that the accessing medications like morphine was a big challenge for inpatient service. One of the key informant interviews revealed that:

The drug morphine is key to cancer patients. However, the drug was not available in the facility for long period of time. It wasn’t in stock for a very long time and now it is easy to say no morphine at all.

Another key informant also responded that affordability issue of required medications is also the major challenge for patients.

*“Medicines are affordable only for those with health insurance. If you don’t have insurance*,* you can’t buy them here. We buy our medicine from outside because we don’t have health insurance. If the hospital sold the medicine to us at their prices*,* it would make a huge difference compared to buying it outside. Medicine is very expensive outside the hospital*,* but it’s much cheaper here. It’s great for those with insurance because they get a discounted price. “*.

*These findings were supported by previous studies done in Ethiopia* [28, 43].

Most of the respondents (83.6%) agreed that they were informed about common neuropsychiatric issues, got communicated about their treatment, and discussed on their diagnosis and prognosis with palliative care professional. The IOM also recommended that patients be provided with clear, accurate, and understandable information regarding all facets of their care, including the diagnosis, prognosis, and course of treatment [40]. In the same way, majority of the respondents (87.4%) perceived that their friends and families were involved in decision-making and the palliative care provided in the hospitals was family-centered and coordinated. This finding was lower than the study done in Singapore, in which 92.5% perceived that their care givers were involved [44]. The possible explanation for this gap could be due to difference in sample size and study setting.

Our study found that patients aged 30–39 years more perceived that the palliative care they received was patient centered. This may be due to patients in this age group had better health literacy compared to the older age group. The finding is supported by the study done in South Wollo, which specifies a difference in patient-provider interaction between adults and younger patients [45]. Patients who were divorced were less likely to perceive the palliative care service as patient-centered compared to those who were married. The possible reason for this may be due to patients who are divorced may not have someone to take care of them during treatment, which may affect their perception negatively [46]. The study also showed that patients whose monthly income greater than 12,000 ETB were less likely perceived that the palliative care provided for them was patient centered. This could be because patients with higher income have higher expectations and demands compared to those with a lower income. This could affect their judgment of the service’s quality as it could be biased by their expectations. Cancer patients who understand their health condition more perceived that the palliative care service was patients centered as compared to their counters. The reason may be due to patients with low health literacy may not have an easy communication, which can affect their perception of a patient-centered care [47]. The finding is supported by the study conducted in Bahir Dar city that patients who didn’t have information about their condition were 2.88 more likely to perceive the service was patient-centered [28]. Patients who had no intimate relation with the care providers were perceived that the palliative care they received was patient centered as compared to their counters. This could be explained as patients who are not intimate with their provider may not have trust on them. Perceived easiness of the service also negatively affected the experience of patient centered care. The reason may be due to patients accessing the services easily might enable to get timely care and address patients’ and their family needs. Timely access to healthcare is one core component of a patient-centered care, according to the Picker’s Institute [48]. Long appointment time was also negatively affected the perception of patients on patient centered palliative care. This may be due to that as the appointment time become long, patients may face complications and suffering more. Our qualitative finding revealed this as due to the large number of patients, there are long queues to get an appointment and this may increase the chance of having serious complications among cancer patients.

### Limitation and strength of the study

The study employed mixed method approach and use multiple data sources to triangulate and support findings. However, the study may have social desirability bias which overestimate the result. To minimize this bias the data collectors assured about the objective of the study and their negative feedbacks wouldn’t affect them and the treatment they received.

### Policy implications

This study generated empirical evidence on the gaps, challenges and factors of existing palliative care services. The study can help policymakers design more effective, patient-centered health policies that improve cancer patients’ quality of life and overall healthcare outcomes. The study revealed key patient preferences and needs, guiding the development of national palliative care guidelines that prioritize holistic, compassionate care.

## Conclusion and recommendations

The magnitude of patient-centeredness care for adult cancer patients in palliative care was found to be 77.9%. Majority of the respondents perceived that the palliative care service providers respected them and their families, the palliative care service was integrated, their physical and emotional comfort was maintained and their families and themselves were involved in decision making of their health condition. However, only one-third of them responded that accessing the services like laboratory results and medications were easy. Age of the patients, marital status, monthly income, participation in decision making, intimacy with the provider, ease of access of the services, and length of appointment time were significantly associated factors with the patient-centeredness of the adult cancer patients’ palliative care service.

The authors recommend that hospitals should streamline service delivery, particularly by enhancing coordination between departments. Introducing dedicated staff or systems to track and expedite lab results and prescriptions could improve access. It is also better to implement strategies to minimize appointment waiting times. This could include optimizing scheduling systems and increasing the availability of healthcare providers. Healthcare providers should actively involve patients and their families in treatment discussions, improving communication and encouraging shared decision-making. To enhance patient-centered care, providers should develop stronger emotional connections with patients, fostering trust and comfort. Regular communication, empathy, and psychological support could contribute to this intimacy.

Recognizing the influence of marital status, tailored emotional and social support should be offered, especially to single or widowed patients, ensuring they receive the care and attention necessary to compensate for reduced family involvement.

## Data Availability

Data will be provided from the correspondent author based on reasonable request.
